# Electrochemistry of Cobalta Bis(dicarbollide) Ions Substituted at Carbon Atoms with Hydrophilic Alkylhydroxy and Carboxy Groups

**DOI:** 10.3390/molecules27061761

**Published:** 2022-03-08

**Authors:** Lukáš Fojt, Bohumír Grüner, Jan Nekvinda, Ece Zeynep Tűzűn, Luděk Havran, Miroslav Fojta

**Affiliations:** 1 Department of Biophysical Chemistry and Molecular Oncology, Institute of Biophysics of the Czech Academy of Sciences, Královopolská 135, 612 65 Brno, Czech Republic; raven@ibp.cz (L.H.); fojta@ibp.cz (M.F.); 2 Department of Synthesis, Institute of Inorganic Chemistry of the Czech Academy of Sciences, Hlavní 1001, 250 68 Řež, Czech Republic; gruner@iic.cas.cz (B.G.); nekvinda@iic.cas.cz (J.N.); tuzun@iic.cas.cz (E.Z.T.)

**Keywords:** metallacarborane, cobalta bis(dicarbollide) ions, glassy carbon electrode, differential pulse voltammetry

## Abstract

In this study we explore the effect on the electrochemical signals in aqueous buffers of the presence of hydrophilic alkylhydroxy and carboxy groups on the carbon atoms of cobalta bis(dicarbollide) ions. The oxygen-containing *exo*-skeletal substituents of cobalta bis(dicarbollide) ions belong to the perspective building blocks that are considered for bioconjugation. Carbon substitution provides wider versatility and applicability in terms of the flexibility of possible chemical pathways. However, until recently, the electrochemistry of compounds substituted only on boron atoms could be studied, due to the unavailability of carbon-substituted congeners. In the present study, electrochemistry in aqueous phosphate buffers is considered along with the dependence of electrochemical response on pH and concentration. The compounds used show electrochemical signals around −1.3 and +1.1 V of similar or slightly higher intensities than in the parent cobalta bis(dicarbollide) ion. The signals at positive electrochemical potential correspond to irreversible oxidation of the boron cage (the C2B9 building block) and at negative potential correspond to the reversible redox process of (CoIII/CoII) at the central atom. Although the first signal is typically sharp and its potential can be altered by a number of substituents, the second signal is complex and is composed of three overlapping peaks. This signal shows sigmoidal character at higher concentrations and may be used as a diagnostic tool for aggregation in solution. Surprisingly enough, the observed effects of the site of substitution (boron or carbon) and between individual groups on the electrochemical response were insignificant. Therefore, the substitutions would preserve promising properties of the parent cage for redox labelling, but would not allow for the further tuning of signal position in the electrochemical window.

## 1. Introduction

The bis(icosahedral) cobalta bis(dicarbollide)(1-) ion, discovered by M. F. Hawthorne in 1965 [1,2,3,4,5], contains two η^5^ coordinated dicarbollide ligands that sandwich a central Co(III) atom [4,5,6]. Due to its unique properties, such as aromaticity [7], space-filling properties, high thermal and chemical stability, low toxicity, hydrophobic interactions due to the hydridic character of B-H bonds [4,8,9,10], the formation of dihydrogen bonds [9,11], and easy penetration across phospholipid bilayers [12,13,14,15] and cell membranes [15,16,17,18,19,20], this ion has been applied in different areas of contemporary materials research [4,21] and medicinal chemistry [4,22,23,24]. The scope of potential applications in drug design includes enzyme inhibitors such as HIV-protease [25,26,27], carbonic anhydrase IX [16,28,29], anticancer compounds [16,29,30], antibiotics and antimycotics [31,32,33,34,35] and components for modulating the hydrophobic interactions of biomolecules [36,37,38].

The electronic structure in the inner orbital corresponds to a fully delocalized aromatic system [7], and along with the presence of a Co(III) central atom, generates interesting electrochemical properties [39]. Previous studies [24,39,40,41,42,43] and our recent results [44] suggest that the cobalta bis(dicarbollide) ion can be considered a good candidate for the tunable electrochemical labelling of biomolecules. Indeed, some icosahedral metallacarboranes have already been used as redox labels for the electrochemical detection of biomolecules [45,46]. The cobalta bis(dicarbollide) ion has been used in bioconjugation studies [3,4,40,41]. In addition, a study on the closely related ferra bis(dicarbollide) ion appeared recently in the literature [40]. According to the published results, derivatives of cobalta bis(dicarbollide) proved to have favorable electrochemical properties when modified boronated nucleotides were studied alone [44] and when such building blocks were incorporated into DNA [40]. The design of all these systems is based on the cleavage of cyclic ether rings bound to the B(8) boron atom of the cobalta bis(dicarbollide) ion by a nucleophilic attack of amino functions on the α-carbon position adjacent to the oxonium atom of the ring [3,4,41,47]. This truly universal chemical method, which may be called the “boron click approach”, was first reported by Plešek in 1991 [48]. Thus, only compounds substituted on boron atoms have hitherto been studied. The boron cluster is attached to the biomolecule by a six-atom-long linker terminated by a protonated ammonium group. This has some further implication, not due to the chain length, but rather due to the presence within it of three donor atoms. This type of ethyleneglycol connection is susceptible to forming crown-ether-like arrangements with sodium ions (or K^+^ and Ca^2+^ ions) [49,50]. This results in increased hydrophobic properties and a tendency to self-assemble into micelles [51] or undergo compartmentation in solution. It is also well known from carborane chemistry that simple protonatable groups on boron atoms have distinctly different physicochemical properties than those on carbons.

Herein we therefore focus on a new alternative system based on carbon substitutions, presenting the first electrochemical screening study over a series of compounds containing hydroxyalkyl [52] and carboxyl [53] groups on the cobalt bis(dicarbollide) ion. This is possible only now as synthetic pathways to these substitutions have appeared only recently. In general, -COOH, -SH, and -OH groups attached on carbon atoms in icosahedral carboranes [4,54] are distinctively more acidic and better comparable in properties to organic compounds than those on boron atoms. The compounds selected for this study thus contain polar oxygen atoms in terminal units, which, according to our previous results on other clusters [55,56,57,58], increased the solubility of boron compounds in phosphate buffers and aqueous media and improved electrochemical response. The selection was also made with the aim of using these compounds as biolabels. Herein we compare the electrochemical properties of carbon-substituted derivatives with similar boron-substituted compounds available by contemporary synthetic routes, which contain methoxy groups at B(8,8′) sites [42,59]. All compounds provided an electrochemical response that was sufficiently distinct from typical windows for biomolecules. The results are a first step in the development of direct site-specific labelling of biomolecules, following in situ bioconjugation schemes using known methods. Thus, the -COOH group could be used for attachment to a biomolecule by an established procedure of ester bond [60] or amide bond formation, or procedures for esterification reactions when considering the studied alcohols.

## 2. Results and Discussion

**Figure 1:**
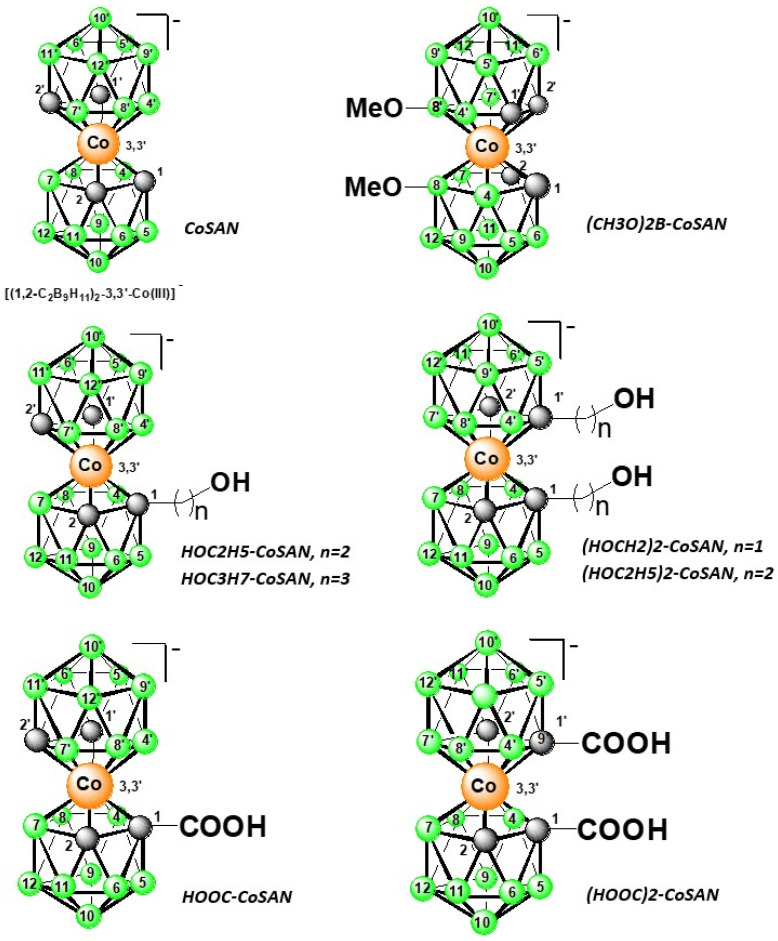
Schematic structural formulas of the metallacarboranes and all used *exo*-skeletal substituents. For all cases, black ball—CH group; green ball—BH group.

All samples were sandwich complexes consisting of a central cobalt atom coordinated between two C_2_B_9_ ligands. The samples were modified by different *exo*-skeletal substituents. Within this series, the substituents comprised alkylhydroxy or carboxy residues. The systematic formulae of metal bis(dicarbollide) ions salts investigated in this study are given in Table 1. For the structural formulae, please see Figure 1. In contrast to our previous results obtained with metallacarboranes and their *exo*-skeletal derivatives [44], all *exo*-skeletal substituents used in this study were bound to the carborane cage at the carbon atom (previously only at the boron atom). Our aim was to determine to what extent the presence and position of a hydrophilic substituent can alter the electrochemical behavior of metallacarborane derivatives. This would reflect differences of electronic densities resulting from the contribution of substituents attached at carbon or boron atoms.

### 2.1. Electrochemical Behavior of Hydroxy- and Carboxy-Substituted Metallacarboranes

**Figure 2:**
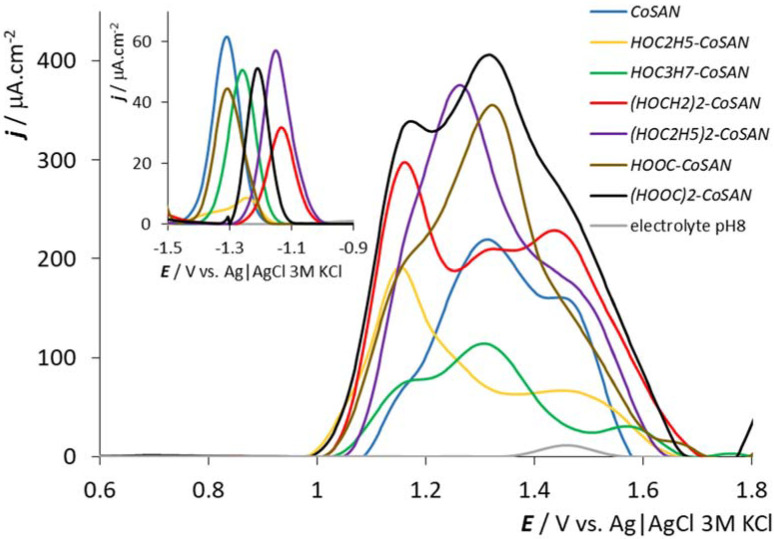
DPV of all used compounds, PB, pH = 8, 500 μM concentrations of all samples, GCE. See caption in the panel for sample identification.

**Figure 3:**
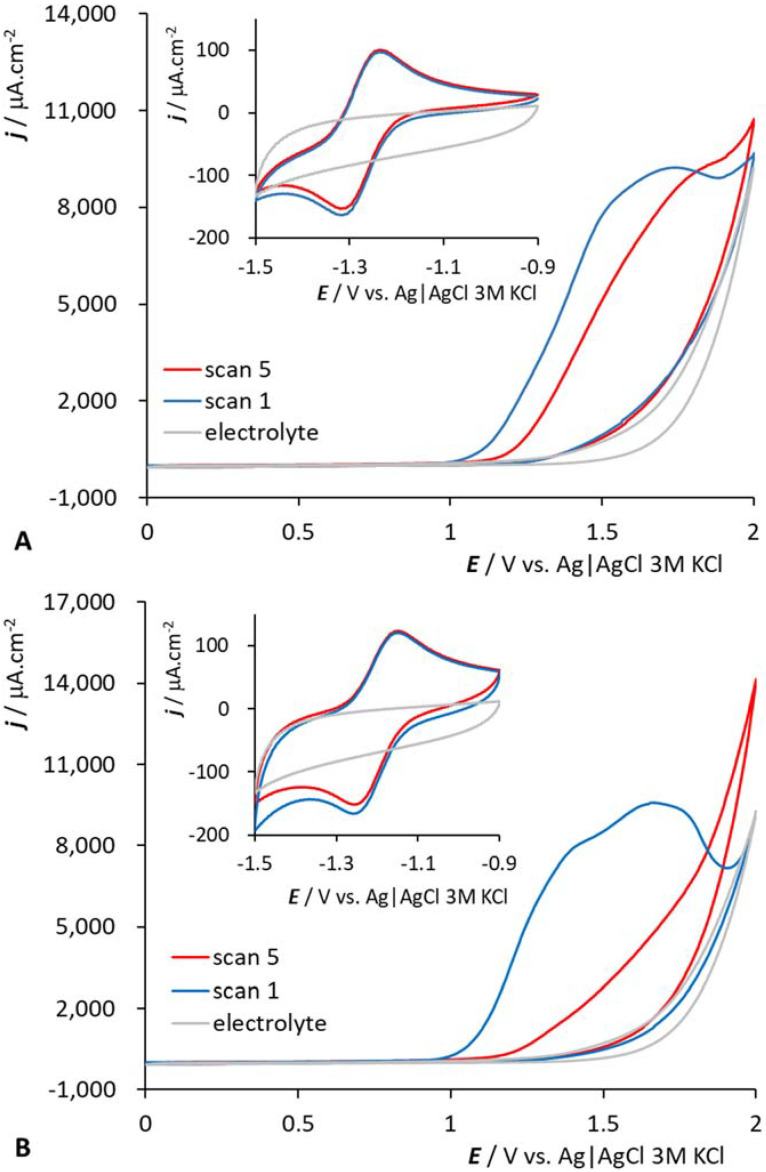
Cyclic voltammogram of *HOOC-CoSAN* (**A**) and *(HOOC)2-CoSAN* (**B**) in PB, 1000 μM concentration, GCE, pH = 8, scan rate ν = 100 mV·s^−1^.

The electrochemical behavior of all measured samples is displayed in Figure 2. The electrochemical data, systematic formulae and abbreviations used in the text are presented in Table 1. All compounds studied give a single symmetrical peak in the negative part of the potential window of the used GCEs. This peak is ascribed to the Co(III/II) redox process. We can observe oxidation (the measurement starts at E = −1.7 V) after corresponding electrochemical reduction to Co(II). According to the CV measurements (see Figure 3 for the *HOOC-CoSAN* and (*HOOC)2-CoSAN*, the situation for the other samples is similar, see Figure S1 in ESI. We can deduce the reversible character of this negatively situated electrochemical response (the scan rate–peak height dependence (not shown) suggest that the electrochemical reaction is driven by diffusion).

The peak position for the reversible electrochemical reaction has an interesting trend for all measured samples: the peak height is similar for almost all samples, about 50 μA·cm^−2^, except for the ill-developed peak for *HOC2H5-CoSAN*. Starting at E = −1.30 V for the parent *CoSAN* and a similar value for the simplest substitution, *HOOC-CoSAN*, the compounds with a single *exo*-skeletal substituent exhibit generally positively shifted electrochemical oxidation potential. This continues to grow up to E = −1.10 V for the disubstituted dicarboxylic acid and dialkylhydroxy derivatives *(HOCH2)2-CoSAN* and *(HOC2H5)2-CoSAN*. The reason for this shift can be seen in the perturbation of the electronic and geometric structure of the parent *CoSAN* by the hydrophilic substituents, which seem to facilitate the electrochemical reaction.

However, the situation in the positive part of the potential window is completely different. In accordance with our previous observations [44,55,56,57,58], the electrochemical response is irreversible in this region and could be ascribed to the electrochemical oxidation of the carborane cage in the structure of the metallacarborane sandwich (in this case the two C_2_B_9_ dicarbollide ligands).

If the *CoSAN* is considered as a template, we can observe one main broad peak situated at E = +1.32 V. Focusing on the negative potential, a small shoulder can be observed. Moving up to positive potentials, one overlapping peak appears at a potential of around E = +1.45 V, which is near to the potential of the electrode–electrolyte surface reaction [44] (see the peak for the electrolyte at the same potential). All studied samples exhibit similar, but quite complex electrochemical behavior. The electrochemical signals comprise three overlapping peaks situated at similar potentials. Generally, a similar line shape is observed for samples with identical substituents that differ in their numbers (most clearly for *(HOOC)2-CoSAN* and *HOOC-CoSAN*). However, peak height and peak separation differ markedly. The reason for this behavior may be similar to the situation at the negative potential part of the electrochemical window. As is known from the literature [61], the cobalta bis(dicarbollide) ion has three energy minima, resulting in discrete rotamers—cisoid, transoid and gauche—identified by chemical calculations and found in solid-state structures [2,3,4]. The three peaks may thus originate in energy minima corresponding to the higher stability of the three different rotamers. As expected, the terminal hydroxy and carboxy groups may be involved in hydrogen bonding, and furthermore, the longer chains may contribute to steric clashes that may a play role in the distribution of different rotamers within a time scale, which could be reflected in the position of observed maxima and the shape of electrochemical responses. This hypothesis is further supported by the recent electrochemical study of *CoSAN* substituted with a rigidifying bridge interconnecting two C_2_B_9_ ligands and that led to the induction of a conformationally restricted cisoid conformation. Unlike the compounds presented herein, those compounds showed only one major electrochemical peak in the respective range [62]. Furthermore, even if it is known that the speed of rotation is quite high in solution (as described recently for the closely related iron sandwich) [63], the situation at the electrode interface is different. The compounds are slightly stabilized when accessing the electrode surface, where often at least slight adsorption takes place (which may hinder the rotation feasibility). Secondly, the compounds are asymmetrically substituted and correspond to racemic mixtures. The electrochemistry represents an average over all forms of molecules; thus, we are able to observe all three rotamers simultaneously. The reason for the shift in electrochemical potential may lie in the different accessibility of the electrode surface to boron and carbon atoms in given rotamers. Coming out from our previously published results on metallacarboranes and their *exo*-skeletal derivatives [44,56] and newly obtained knowledge, we can assume that higher potential shift and/or the emergence of new peaks strongly depends on the physicochemical properties of the *exo*-skeletal substituents. In the case of *CoSAN*, a significant oxidation peak shift occurs in the case of iodine and chlorine *exo*-skeletal substituents [44] (iodine has bulky valence orbitals, chlorine has ahigh electronegativity). The present results corroborate these findings.

### 2.2. pH Dependencies of Selected BCCs

**Figure 4:**
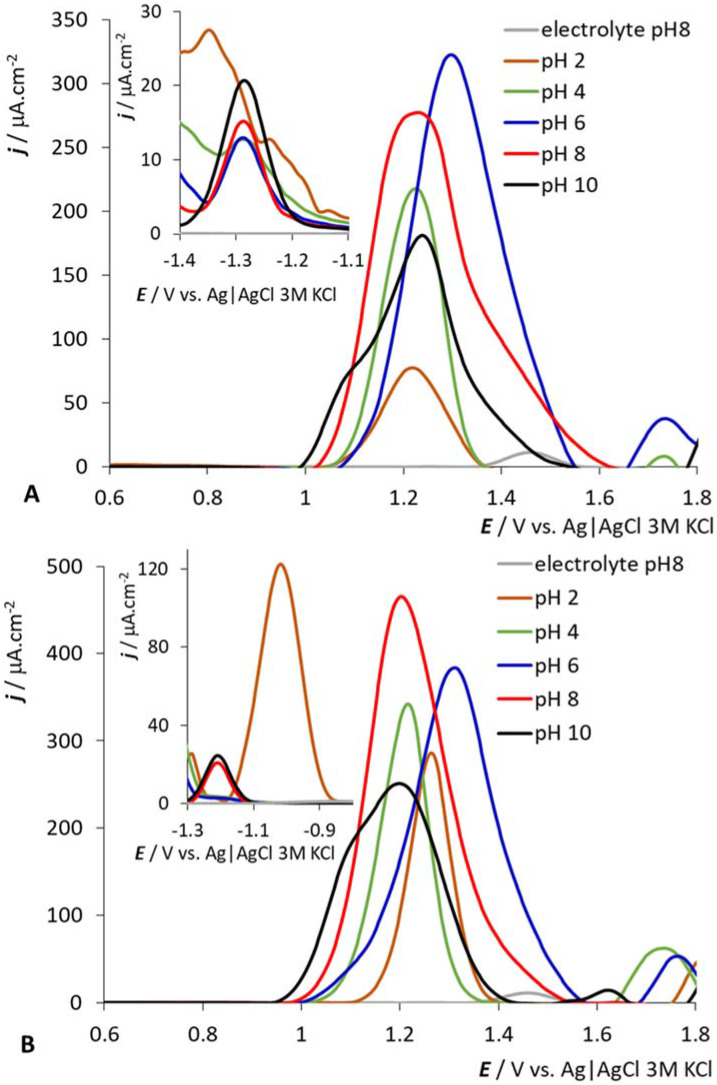
pH dependencies of 200 μM *HOOC-CoSAN* (**A**) and *(HOOC)2-CoSAN* (**B**), GCE. See the captions in the panels for pH value identification.

The pH dependence of selected anions is shown in Figure 4; the results for samples at 200 μM concentration are presented and discussed. We selected samples with one and two carboxylic groups for this study, which sit directly on the carbon atom and can be easily protonated/deprotonated—compounds corresponding to *(HOOC)2-CoSAN* and *HOOC-CoSAN*. The situation is simple at the positive potentials (the region of the electrochemical oxidation of dicarbollide ligands). For both selected samples, only one dominant peak is observed. *HOOC-CoSAN* has peak height maxima for pH = 6, *(HOOC)2-CoSAN* for pH = 8. At lower and higher pH, the peak height gradually decreases with only a small shift in the observed potentials. The situation for *HOC3H7-CoSAN* and *(HOC2H5)2-CoSAN* is similar, and differs for *(HOCH2)2-CoSAN* and *HOC2H5-CoSAN*—for the three highest pH values (6, 8 and 10) we can observe the same peak heights, but with a shift of electrochemical potential (to more positive values with the decrease of pH; see Figure S2 in ESI). In the negative potential region of the Co(III) redox process, we can discern a similar trend for all samples. At pH = 2, the signal becomes distorted or highly elevated. Both these effects have their origin in the protonation of the groups and the formation of hydrogen bonds. This may result (in dynamic time scales) in a slower rotation of ligands or in partially restrained conformation, particularly in the case of disubstituted species, where a formation of bridging -OH-H_3_O^+^-HO- arrangement between two dicarbollide ligands can be expected.

### 2.3. Concentration Dependence

**Figure 5:**
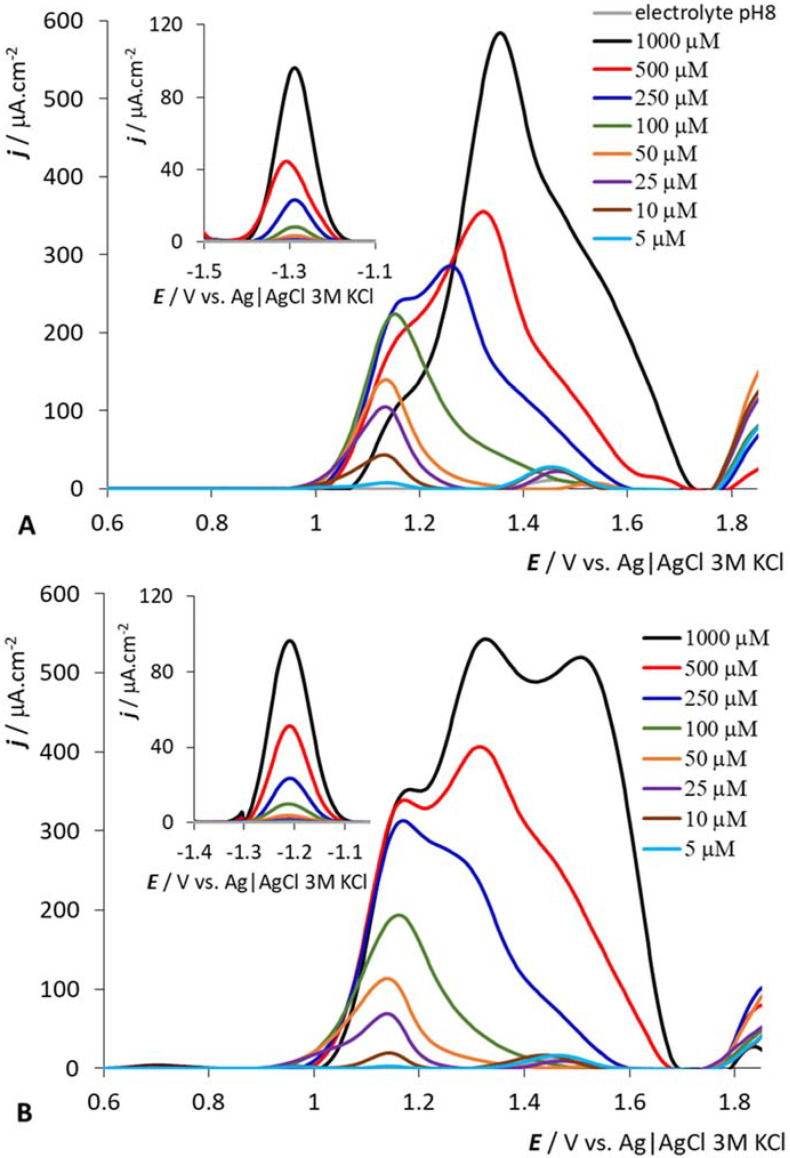
Concentration dependencies of *HOOC-CoSAN* (**A**) and *(HOOC)2-CoSAN* (**B**), at pH 8, GCE. See the captions in the panels for concentration identification.

The results (Figure 5 and Figure S3 in ESI) show a similar trend for all samples, with the exception of HOC2H5-CoSAN. For higher concentrations, sigmoidal types of dependence are generally observed at the positive potential area of the polyhedral carborane cage oxidation; for higher sample concentrations, the signal does not increase linearly. This behavior has already been observed in other metallacarboranes [44]. Based on current and previous results we can assume that these effects may originate in the tendency of the metallacarborane anions to aggregate in aqueous solution. This behavior is well known from recent studies using other techniques [49,51], which further support the electrochemical results. In the positive potential area and at high concentrations (approximately above 250 μM), the predominant peak is located at approximately +1.30 V. When the concentrations decrease, this peak slightly decreases, and its maximum is transformed into another peak located around +1.15 V. The exception is HOC2H5-CoSAN, where the peak at +1.15 V remains at the same potential even after increasing the concentration to 1 mM (see ESI, Figure S3). When considering the emerging potential of metallacarboranes in medicine and biochemistry [8,27,39], rapid information about aggregation and/or micelle formation in water-based media is of particular concern. From the results described in this paragraph, it follows that electrochemical methods can provide a new tool for easy inspection if a substituted boron anion tends to aggregate in aqueous solution under particular experimental conditions. In particular, this provides for a ready estimation of the concentration at which the formation of aggregates or micelles starts to occur.

Interestingly, the concentration dependence in the region of the Co(III) redox process, on which most electrochemical studies are centered, does not follow this trend and the dependence on concentration is almost linear. Indeed, as can be expected, the reversible reaction may be not so highly affected by this behavior. Therefore, only the signal of the irreversible oxidation of the carborane polyhedral can be used for diagnostics of the aggregation phenomena. On the other hand, this signal has a complex character and its deconvolution is needed.

### 2.4. Comparison of Different Substituent Positions (B or C Atom)

**Figure 6:**
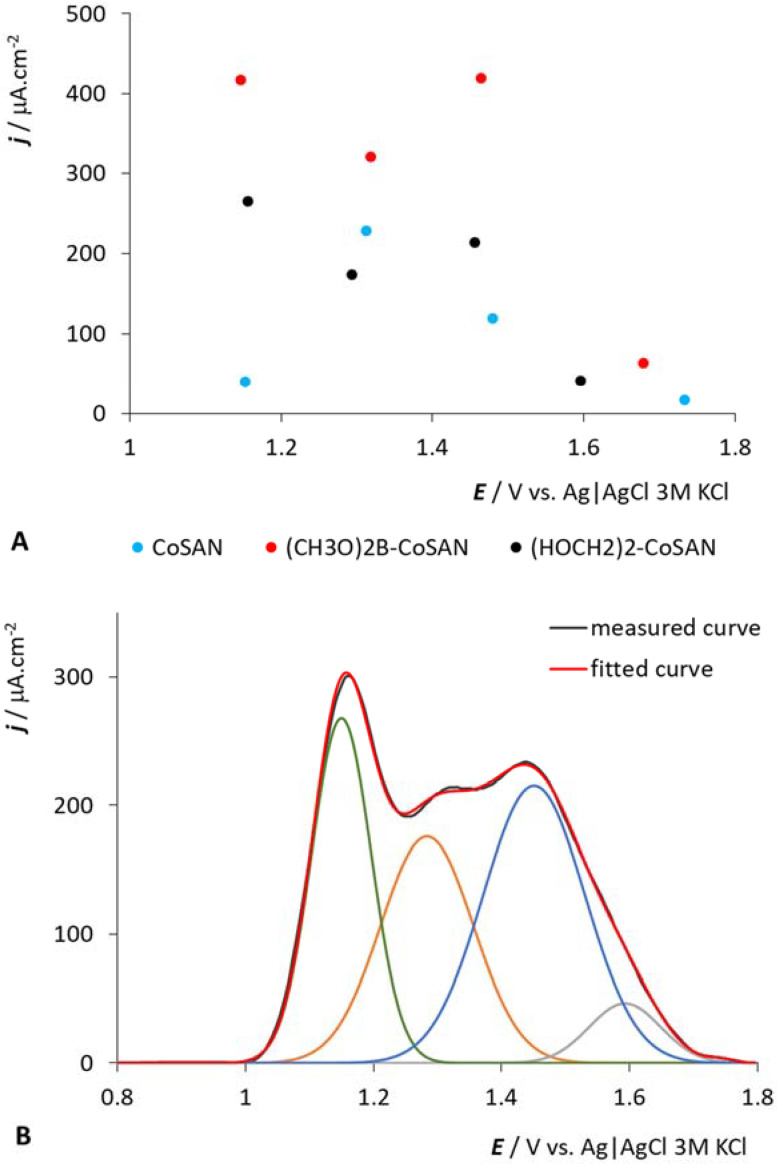
Deconvolution results (peak heights and position of fitted peaks) for the *CoSAN*, *(HOCH2)2-CoSAN* and *(CH3O)2B-CoSAN* [44] (**A**). Example of measured, sum of fitted peaks and individual fitted peaks for *(HOCH2)2-CoSAN* (**B**). All fitted curves were measured in PB of pH = 8, 500 μM concentration.

It is reasonable that the most important changes in electron density can be introduced by substitution at the carbon and boron atoms in ligand planes that bind in η-fashion to the central cobalt atom in *CoSAN* polyhedra. We compare here the compound with two (HOCH_2_)_2_ substituents on the carbon atom *(HOCH2)2-CoSAN* with a similar compound that has two (O-CH_3_)_2_ groups on boron B(8,8′) sites in *(CH3O)2B-CoSAN*. The latter compound should be taken for comparison due to the limited progress of the cage modification. Unfortunately, no fully identical congeners that would have the same substitution on the B or C atom are currently available by synthesis. The electrochemical responses of these two samples in the positive potential area are complex (see Figure 2 and Figure 6) and consist of several overlapping peaks. For this reason, we used Fytik software for the deconvolution of the electrochemical curves in this potential region. The deconvolution was performed using a Gaussian distribution with an average fit error of 10%. For better clarity we also included the electrochemical response of the parent *CoSAN*. The results are displayed in Figure 6. Surprisingly enough, there is no distinguishable difference between those two substitutions except for the peak height in the range of positive potentials ascribed to cage oxidation. The character of the peak is quite complex, and is referred to in the discussion in Section 2.1. The deconvolution provided four peaks for each compound, in which two positive peaks are only slightly shifted in the case of disubstituted compounds; only the most positive signal significantly differs in its position. Nevertheless, this peak might originate from the electrode surface–electrolyte reaction and cannot be considered sufficiently reliable, as mentioned above. Thus, the potential of the fitted peaks does not change, only the peak heights. The corresponding number of the three peaks may give indirect evidence that the peaks are associated with the presence of rotamers. This is because the B(8)-H bond with the highest electron density known to be easily activated in an EINS-type reaction [2] cannot be involved in the redox process in (*CH3O)2B-CoSAN*. If the complex character of the signal is associated with the three most electron-rich sites H-B(8,9,12), the deconvolution would inherently provide a simpler pattern for *(CH3O)2B-CoSAN* because most of the reactive sites B(8,8′) are blocked by substitution.

### 2.5. General Remarks

Herein we present the electrochemistry of hydroxy and carboxy *exo*-skeletal derivatives of CoSAN. Present knowledge on the electrochemistry of metallacarboranes indicates that the responses at the positive potentials could be denoted as the electrochemical oxidation of the core carborane structure. The peaks located in the negative potential part are connected with the reversible redox processes of the central Co atom. Our results indicate that the oxidation of the polyhedral boron cage is completely irreversible. Unfortunately, the electrochemical methods that would allow for the theoretical evaluation of these processes are available only for reversible or quasi-reversible reactions (e.g., the Pourbaix diagrams). Therefore, the mechanism of the studied compounds’ electrochemical responses cannot be understood fully. The reason for the uncommon electrochemical behavior of the boron cluster compounds may lie in their specific electronic properties. Considering the irreversible oxidation process, the electronic density is probably removed from the B-H [44]. Due to electron delocalization over the boron cages, we can assume that the exact place of the electron exchange cannot be defined.

## 3. Materials and Methods

### 3.1. Synthesis

All compounds used in this study were prepared in accordance with previously published procedures. The carbon-substituted hydroxyalkyl derivatives of the general formula [(1-HO(CH_2_)_n_-1,2-C_2_B_9_H_10_)(1′,2′-C_2_B_9_H_11_)-3,3′-Co)]Cs and [1,1′-(1-HO(CH_2_)_n_-1,2-C_2_B_9_H_10_)_2_-3,3′-Co] were prepared by low-temperature lithiation reaction of the Cs[(C_2_B_9_H_11_)2-3,3′-Co] with BuLi in DME followed by reaction with *para*-formaldehyde, oxirane or trimethylene oxide [52]. The products were isolated by chromatography and crystallization. In the case of dihydroxyalkyl compounds, only the racemic diastereoisomer was used in this study, and was isolated according to the procedure described in the literature [16]. The carboxylic acids were prepared by reactions of the carbon lithiated ion with carbon dioxide [53]. The boron-substituted compound was isolated from reaction mixtures obtained from acid-catalyzed reactions of cobalta bis(dicarbollide) with para-formaldehyde, as described in the literature [59]. The compounds were characterized by NMR, MS and HPLC methods, with results matching data published in previous papers [52,53,59].

### 3.2. Electrochemistry

An Autolab 302 potentiostat (Ecochemie, The Netherlands, and now Metrohm) connected to a conventional electrochemical cell with a three-electrode system was used for the electrochemical experiments. A platinum wire (1 mm diameter, purity 99.99%, Safina, Czech Republic) counter electrode and an Ag|AgCl|3 M KCl reference electrode (Metrohm, Switzerland) were used. A glassy carbon electrode (GCE, 2 mm diameter, Metrohm, Switzerland) was used as the working electrode. Differential pulse voltammetry (DPV, pulse amplitude of 25 mV, pulse width of 50 ms, and scan rate of 8 mV·s^−1^) and cyclic voltammetry (step 5 mV, scan rate 100 mV·s^−1^) were used for the measurements. All measurements were performed at room temperature (295 K). DPV curves were baseline corrected before further processing. Current values were normalized to the geometrical surface area of the used electrodes. The GCE was pretreated by mechanical polishing with silicon carbide papers (SiC polishing papers, Struers, Denmark) and the polishing was finalized using 1 μm diamond particles in spray-on Lecloth B polishing cloth (both Leco, St. Joseph, MI, USA). As a final pretreatment step the GCE was sonicated in tri-distilled water. For electrochemical measurements, phosphate buffers (PBs) of various pH values were mixed from NaH_2_PO_4_ and Na_2_HPO_4_. The concentration of phosphate anions was kept at 0.2 M in all solutions. The systematic formulas of metal bis(dicarbollide) ion salts investigated in this study are given in Table 1. Cations in the salts are specified in Table 1, and served for anion precipitation in the last synthesis step, exchanged for sodium cation using Amberlite CG-120 (Fluka) to increase their solubility in water. Samples used in this study were prepared as 1 mM aqueous solutions according to weighting (in some cases, the solubility in water was lower—see Table 1). All other chemicals were purchased from Sigma-Aldrich and were of the highest available purity, and triple-distilled water was used as the solvent.

## 4. Conclusions

This study presents the electrochemistry of different carbon-substituted cobalta bis(dicarbollide) ion derivatives, substituted with groups containing oxygen atoms, in phosphate buffer. Our results indicate that the presence of different substituents containing terminal hydroxyalkyl or carboxylic residues do not markedly alter the high electrochemical response of the parent *CoSAN*, and that some compounds show even slightly enhanced signals. Therefore, these derivatives can be considered good building blocks with groups suitable for the redox labelling of biomolecules. On the other hand, the electrochemical potential in the negative interval corresponding to the reversible Co(III)/Co(II) redox couple seems to be affected more by the increasing number of substituents on the boron cage than by their nature or the site of substitution. A second potential area of electrochemical response corresponding to an electrochemical oxidation of the cage was also observed. This signal is located in the positive range of potentials from +1.1 to +1.3 V and shows complex character composed of four overlapping peaks, as follows from the deconvolution of selected electrochemical records. The number of peaks did not change even in the case of the boron-substituted compound used for comparison. However, the mutual intensities of the three observed peaks indicate pH and concentration dependency. In the first case this is connected with hydrogen bond formation between substituents in the acidic range, which seems to result in partially limited rotation of the ligands around the central atom. The sigmoidal character of the peak observed for higher concentrations in the latter case might be used as a rapid diagnostic tool for aggregation of ions in aqueous solution.

## Figures and Tables

**Table 1:** Peak positions and heights with simple characteristics of used BCCs on GCE, pH = 8, concentration of all metallaborates 500 μM. For the BCCs marked * the highest achievable concentration of 500 μM was used in the electrolyte solution.

	BCC Charge	Label in the Text	MW	Peak Position/V	Peak Height/μA·cm^−2^
*closo*-[(1,2-C_2_B_9_H_11_)_2_-3,3′-Co)]Cs *	1-	*CoSAN*	323.74	−1.30; 1.14; 1.29; 1.45;	121.0; 55.5; 217.0; 160.0;
[(1-HOC_2_H_5_-1,2-C_2_B_9_H_10_)(1′,2′-C_2_B_9_H_11_)-3,3′-Co)]Cs	1-	*HOC2H5-CoSAN*	367.79	−1.24; 1.16; 1.47;	8.7;191.2; 65.7;
[(1-HOC_3_H_7_-1,2-C_2_B_9_H_10_)(1′,2′-C_2_B_9_H_11_)-3,3′-Co)] Me_3_NH	1-	*HOC3H7-CoSAN*	382.83	−1.26; 1.17; 1.30; 1.58;	50.6; 75.9; 114.1; 30.2;
[1,1′-(HOCH_2_1,2-C_2_B_9_H_10_)_2_-3,3′-Co)] Me_3_NH	1-	*(HOCH2)2-CoSAN*	377.73	−1.13; 1.16; 1.38; 1.44;	31.7; 297.9; 209.7; 228.6;
[1,1′-(HOC_2_H_5_-1,2-C_2_B_9_H_10_)_2_-3,3′-Co)] Me_3_NH	1-	*(HOC2H5)2-CoSAN*	411.74	−1.15; 1.17; 1.26; 1.49;	57.0; 231.8; 375.6; 160.7;
[(1-HOOC-1,2-C_2_B_9_H_10_)(1′,2′-C_2_B_9_H_11_)-3,3′-Co)] Me_4_N	1-	*HOOC-CoSAN*	367.75	−1.31; 1.16;1.32;	44.5; 192.5; 355.6;
[1,1′-(HOOC)_2_-(1,2-C_2_B_9_H_10_)_2_-3,3′-Co)] Me_4_N	1-	*(HOOC)2-CoSAN*	411.76	−1.21; 1.17; 1.32;	51.1; 339.0; 406.5;
[8,8′-(CH_3_O)_2_-(1,2-C_2_B_9_H_10_)_2_-3,3′-Co)] Me4N * [42]	1-	*(CH3O)2B-CoSAN*	377.73	−1.30; 1.15; 1.32; 1.45; 1.68;	94.3; 410.0; 371.0; 377.0; 14.1;

## Data Availability

All data used to support the findings of this study are included within the article and are also available from the corresponding author upon request.

## Supplementary Materials

The following are available online at https://www.mdpi.com/article/10.3390/molecules27061761/s1, Figure S1: Cyclic voltammogram of *HOC2H5-COSAN*, *HOC3H7-COSAN*, *(HOCH2)2-COSAN* and *(HOC2H5)2-COSAN (D)* in PB, 1000 μM concentration, GCE, pH = 8, scan rate ν = 100 mV·s^−1^. Figure S2: pH dependencies of *HOC2H5-COSAN*, *HOC3H7-COSAN*, *(HOCH2)2-COSAN* and *(HOC2H5)2-COSAN* for 200 μM concentration, GCE. Figure S3: Concentration dependencies of *HOC2H5-COSAN*, *HOC3H7-COSAN*, *(HOCH2)2-COSAN* and *(HOC2H5)2-COSAN* at pH 8, GCE.

## Sample Availability

Samples of the compounds are available from the authors.
