# Supplementary material for: Electrochemistry of Cobalta Bis(dicarbollide) Ions Substituted at Carbon Atoms with Hydrophilic Alkylhydroxy and Carboxy Groups
Source: Molecules. 2022 Mar 8;27(6):1761. doi: 10.3390/molecules27061761 (PMC8953506; doi:10.3390/molecules27061761)
Supplement: Supplementary file 1 [file molecules-27-01761-s001.zip › molecules-1622653-supplementary.pdf]

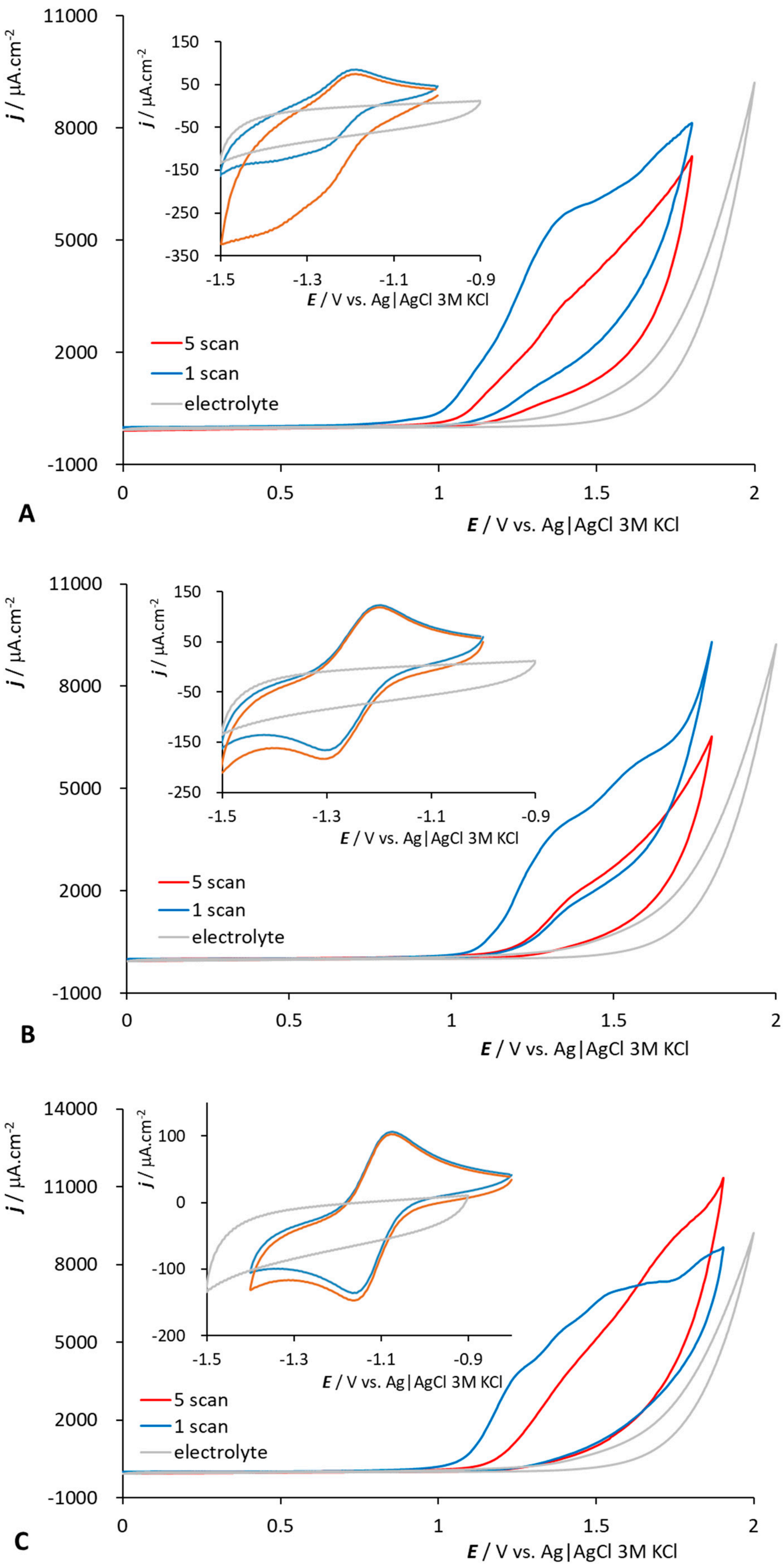

j / µA.cm-2
11000
8000
5000
2000
-1000
0
0.5
1
1.5
2
A
E / V vs. Ag|AgCl 3M KCl
150
50
-50
-150
-250
-350
-1.5
-1.3
-1.1
-0.9
5 scan
1 scan
electrolyte
B
-250
C
14000
100
0
-100
-200

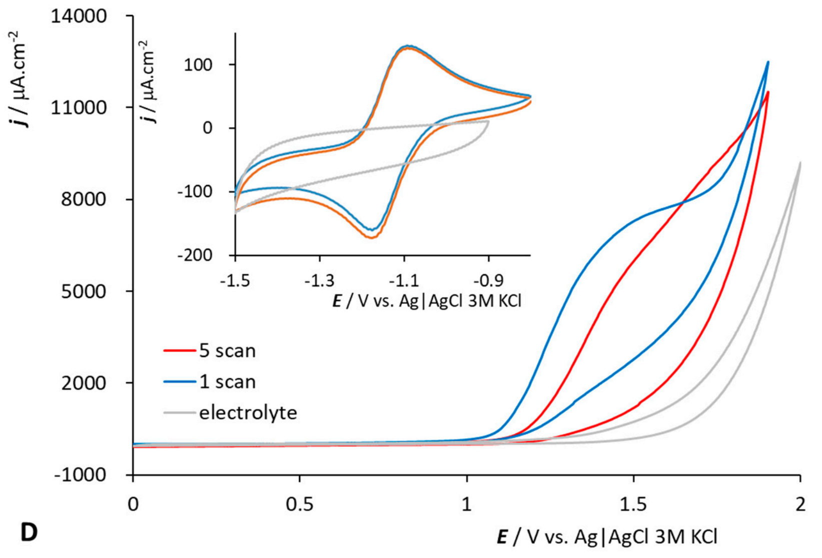

Figure S1. Cyclic voltammogram of *HOC2H5-COSAN* (**A**); *HOC3H7-COSAN* (**B**); *(HOCH2)2-COSAN* (**C**) and *(HOC2H5)2-COSAN* (**D**) in PB, 1000 M concentration, GCE, pH = 8, scan rate = 100 mV.s^{-1}.

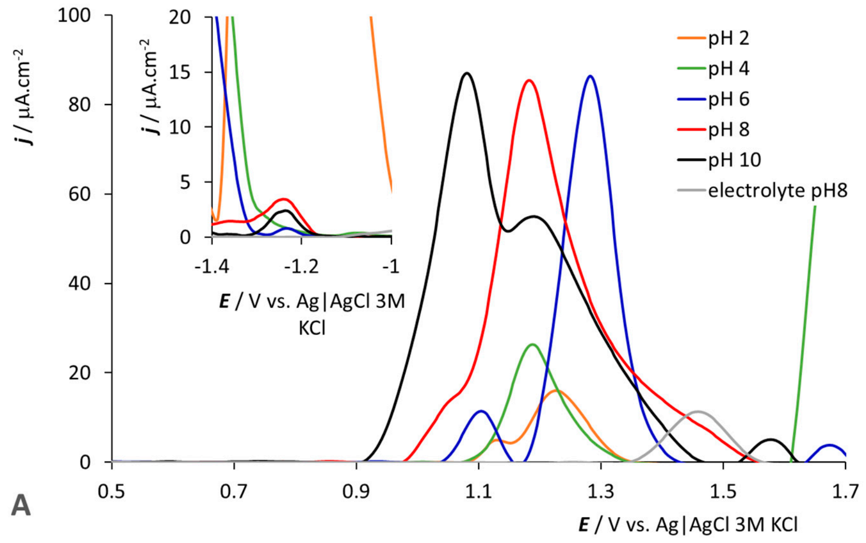

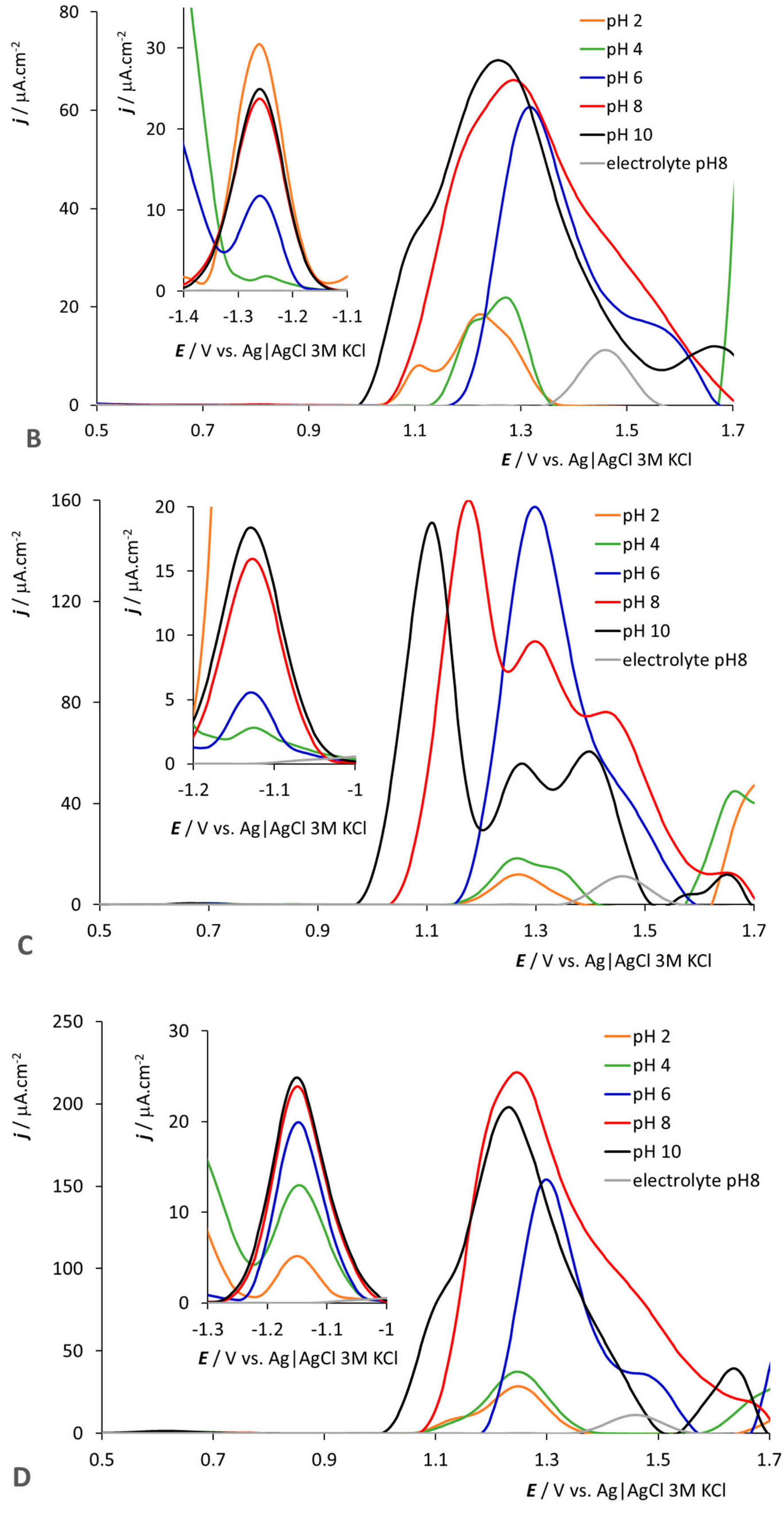

Figure S2. pH dependencies of *HOC2H5-COSAN* (**A**); *HOC3H7-COSAN* (**B**); *(HOCH2)2-COSAN* (**C**) and *(HOC2H5)2-COSAN* (**D**) for 200 M concentration, GCE. See caption in the panel for pH identification.

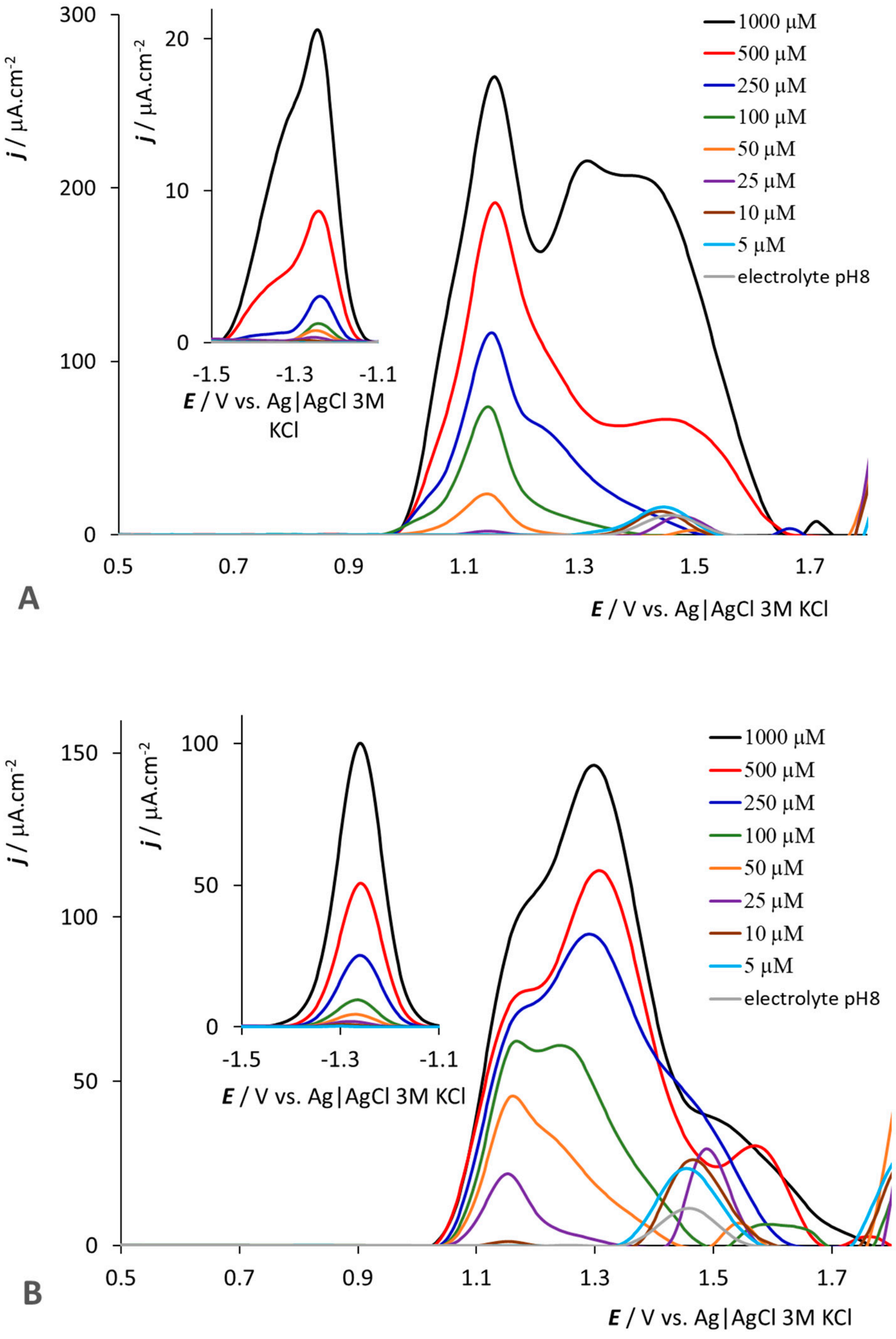

A
j / µA.cm-2
300
200
100
0
0.5
0.7
0.9
1.1
1.3
1.5
1.7
E / V vs. Ag|AgCl 3M KCl
20
10
0
-1.5
-1.3
-1.1
E / V vs. Ag|AgCl 3M KCl
1000 µM
500 µM
250 µM
100 µM
50 µM
25 µM
10 µM
5 µM
electrolyte pH8
B
j / µA.cm-2
150
100
50
0
0.5
0.7
0.9
1.1
1.3
1.5
1.7
E / V vs. Ag|AgCl 3M KCl
100
50
0
-1.5
-1.3
-1.1
E / V vs. Ag|AgCl 3M KCl
1000 µM
500 µM
250 µM
100 µM
50 µM
25 µM
10 µM
5 µM
electrolyte pH8

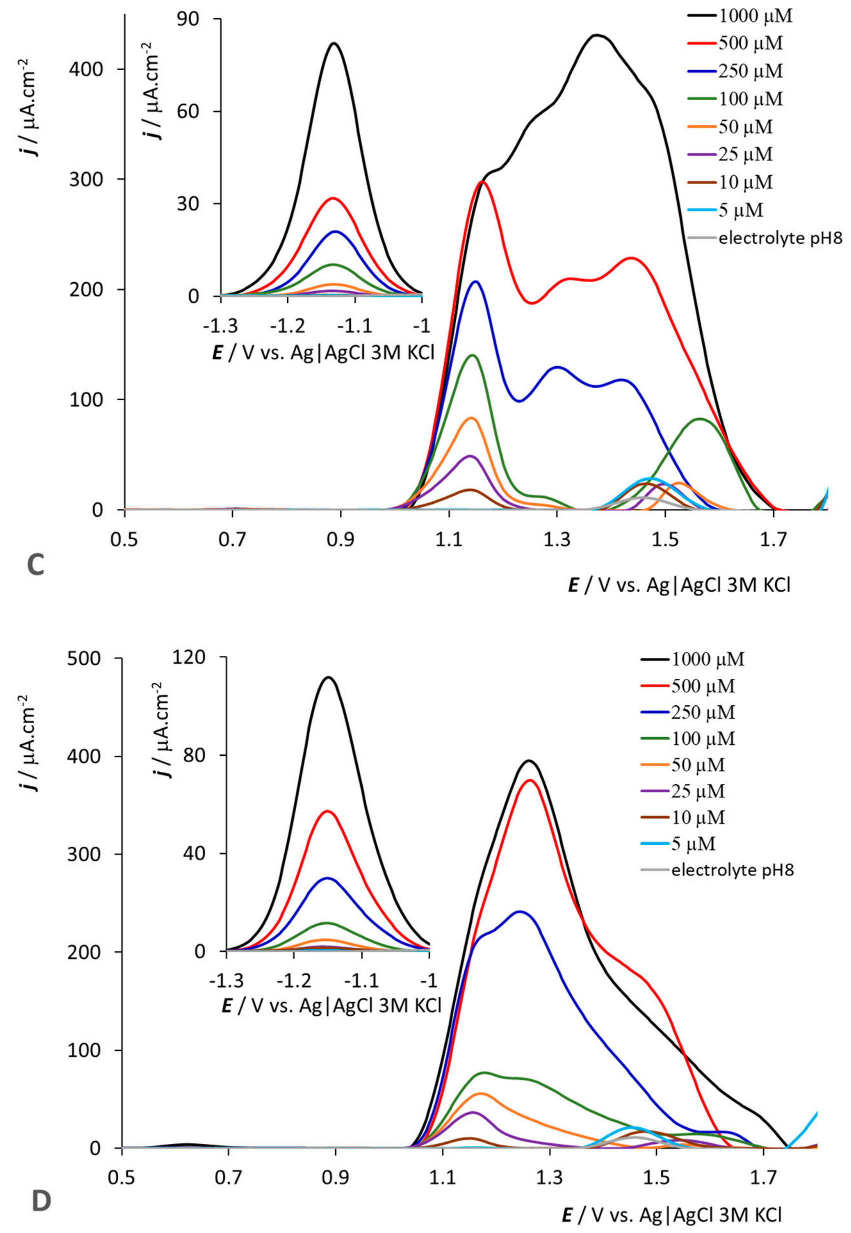

Figure S3. Concentration dependencies of *HOC2H5-COSAN* (**A**); *HOC3H7-COSAN* (**B**); *(HOCH2)2-COSAN* (**C**) and *(HOC2H5)2-COSAN* (**D**) at pH 8, GCE. See caption in the panel for concentrations identification.
